# The Influence of Pituitary Morphology on the Occurrence of Hormonal Disorders in Patients with Empty Sella or Partial Empty Sella

**DOI:** 10.3390/biomedicines13040762

**Published:** 2025-03-21

**Authors:** Bernadetta Kałuża, Mariusz Furmanek, Jan Domański, Aleksandra Żuk-Łapan, Emilia Babula, Iga Poprawa, Małgorzata Landowska, Karolina Jarząbek, Justyna Popczyńska, Paulina Filipowicz, Małgorzata Wielgolewska, Jerzy Walecki, Edward Franek

**Affiliations:** 1 Department of Internal Medicine, Endocrinology and Diabetology, National Medical Institute of the Ministry of the Interior and Administration in Warsaw, 02-507 Warsaw, Poland; 2 Student Scientific Group of the Medical University of Warsaw at the Department of Internal Medicine, Endocrinology and Diabetology, National Medical Institute of the Ministry of the Interior and Administration in Warsaw, 02-507 Warsaw, Poland; 3 Department of Radiology, National Institute of Medicine of the Ministry of the Interior and Administration, 02-507 Warsaw, Poland; 4 Department of Pediatric Radiology, Medical University of Warsaw, 02-091 Warsaw, Poland; 5 Department of Radiology, Center of Postgraduate Medical Education, National Institute of Medicine of the Ministry of the Interior and Administration, 02-507 Warsaw, Poland; 6 Department of Human Epigenetics, Mossakowski Medical Research Centre Polish Academy of Sciences, 02-106 Warsaw, Poland

**Keywords:** empty sella, partial empty sella, primary empty sella, primary partial empty sella, endocrine manifestation, hormonal disorders, arachnoidocele, pituitary, pituitary volume, definition of empty sella, definition of partial empty sella, radiological features of empty sella, radiological features of partial empty sella

## Abstract

**Background/Objectives:** The aim of the study was to prospectively assess the impact of certain parameters of pituitary morphology assessed with the use of magnetic resonance imaging on the occurrence of hormonal disorders in patients with primary partial empty sella (PES) or primary empty sella (ES). **Methods:** Forty-three patients were divided into two groups: group 1—patients with PES (*n* = 20); group 2—patients with ES (*n* = 23). **Results:** Patients with ES were characterized by larger both the transverse (14.8 ± 2.9 mm vs. 17.2 ± 2.9 mm, *p* = 0.016) and anteroposterior (AP) diameters of the pituitary (11.4 ± 1.4 mm vs. 13.2 ± 1.9 mm, *p* = 0.003), a smaller craniocaudal (CC) diameter (3.9 ± 0.62 mm vs. 2.2 ± 0.6 mm, *p* = 0.001), and a lower pituitary volume (332.8 ± 107.6 mm^3^ vs. 243.5 ± 70.9 mm^3^, *p* = 0.001). Moreover, an AP infundibular displacement was more common in patients with ES (7 [35%] vs. 16 [69.6%]., *p* = 0.023). Despite the fact that secondary adrenocortical insufficiency was shown to be significantly more common and ACTH levels to be significantly lower (27.5 ± 13.2 pg/mL vs. 21.8 ± 17.6 pg/mL, *p* = 0.039) in patients with ES (0 [0%] vs. 3 [13.4%], *p* = 0.046), univariate logistic regression did not reveal any significant associations of pituitary diameters, pituitary volume, or pituitary stalk displacement with endocrine disorders, such as secondary adrenocortical insufficiency or hyperprolactinemia, which was confirmed with multivariate logistic regression adjusted for age, sex, BMI, and arterial hypertension. **Conclusions:** Radiologically assessed CC, AP, and transverse pituitary diameters, pituitary volume, or pituitary stalk displacement in patients with PES or ES have no bearing on the rates of hormonal disorders. Nonetheless, certain hormonal disorders may be more common in patients with ES, which suggests a need for hormone-level assessments in this population.

## 1. Introduction

The bulging of the subarachnoid space into the sella turcica results in pituitary compression and enlargement of the sella turcica, which ultimately leads to what is known as empty sella or partial empty sella [1,2,3]. Both empty and partial empty sella may be either primary (possibly associated with a congenital disability in the diaphragma sellae or increased intracranial pressure) or secondary (due to a direct pituitary injury) [1,2,4,5]. The difference between empty and partial empty sella is defined based on radiological features [3]. Partial empty sella is diagnosed when the cerebrospinal fluid occupies less than 50% of sellar volume and pituitary height (i.e., its craniocaudal [CC] diameter) is greater than or equal to 3 mm, whereas empty sella is diagnosed when the cerebrospinal fluid occupies more than 50% of sellar volume and the CC pituitary diameter is less than 3 mm [2,3,6,7]. Despite the fact that the definition of partial empty and empty sella mentions only the CC pituitary diameter and the proportion of the suprasellar cistern filled with the cerebrospinal fluid, pituitary compression by the supracellar cistern may affect other pituitary parameters, such as its volume and its anteroposterior (AP) and transverse diameters, and may result in uneven pressure as evidenced by pituitary stalk indentation or displacement. Despite the fact that primary empty sella or primary partial empty sella is most often an asymptomatic incidental radiological finding [8], in some cases, constituting about 5–30% of the studied population, it may be associated with disorders of more than one hormonal axis and with neurological and ophthalmologic symptoms [8,9]. The risk factors of empty or partial empty sella are better understood than the risk factors of hormone disorders associated with these conditions [3]. The risk factors for partial empty sella or empty sella are known to include sex (female), overweight or obesity, hypertension, high parity, and increased intracranial pressure [3,8,10]. Identifying the specific factors predisposing to the development of hormonal disorders in this patient population may help plan screening tests and standardize guidelines, particularly since, with the growing accessibility of central nervous system imaging, the number of patients with this diagnosis may grow incrementally [3]. Radiological parameters of the pituitary may be such predictors. To date, no clear radiological risk factors for the onset of hormonal disorders have been established in this heterogeneous group of patients. The aim of the study was to assess the radiological parameters of the pituitary and to evaluate the impact of pituitary morphology in patients with partial empty sella or empty sella assessed via magnetic resonance imaging (MRI) for pituitary volume, pituitary CC, AP, and transverse diameters, and the pituitary stalk shape and displacement on the occurrence of hormonal disorders.

## 2. Material and Methods

In order to conduct the study, we analyzed the medical records of the Department of Radiological Diagnostics of the National Medical Institute of the Ministry of the Interior and Administration in Warsaw from the years 2012 to 2022. We identified 891 patients who had undergone an MRI of the central nervous system, 594 of whom had undergone an MRI of the pituitary gland. In 87 patients who had undergone pituitary MRI, morphological evidence of partial empty or empty sella was described. After applying predefined inclusion criteria (age over 18 years, evidence of a primary empty or a primary partial empty sella on pituitary MRI, a follow-up of over 3 years) and exclusion criteria (history of surgical interventions or radiation therapy of the central nervous system, a pituitary or central nervous system tumor, contraindications for MRI of the pituitary gland, lack of consent to participate in the study, hormonal contraception or hormonal replacement therapy), 43 patients were enrolled and had their pituitary re-evaluated on MRI by one experienced radiologist. Following the available definitions, an empty sella was defined as the bulging of the subarachnoid space into the sella turcica in a volume greater than 50%, leading to a pituitary height (i.e., its CC diameter) of less than 3 mm. A partial empty sella was diagnosed when the CC diameter of the pituitary was greater than or equal to 3 mm and the cerebrospinal fluid filled less than 50% of the sellar volume [11,12,13,14].

As a result of this uniform assessment, the patients were divided into 2 groups: Group 1—patients with a confirmed partial empty sella (*n* = 20); Group 2—patients with a confirmed empty sella (*n* = 23).

**Figure 1:**
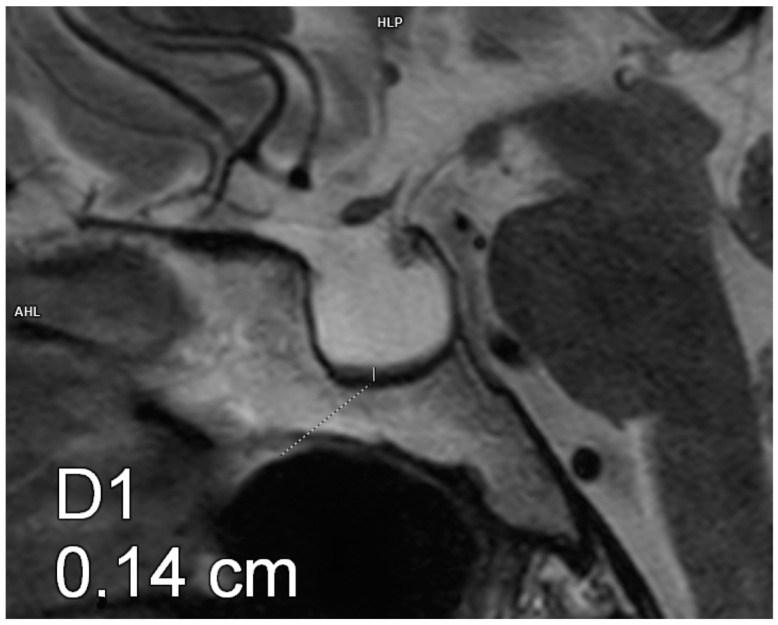
Empty sella. Magnetic resonance imaging, T2-weighted image, sagittal plane. The craniocaudal diameter of the pituitary gland is 1.4 mm.

**Figure 2:**
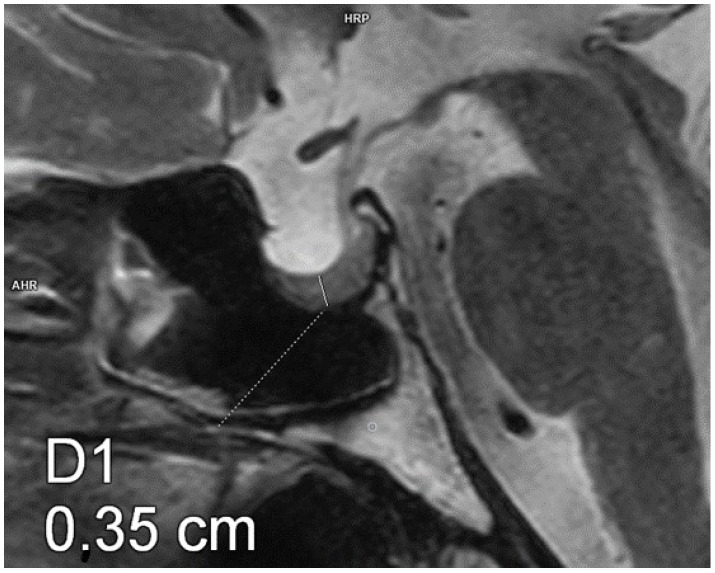
Partial empty sella. Magnetic resonance imaging, T2-weighted image, sagittal plane. The craniocaudal diameter of the pituitary gland is 3.5 mm.

**Figure 3:**
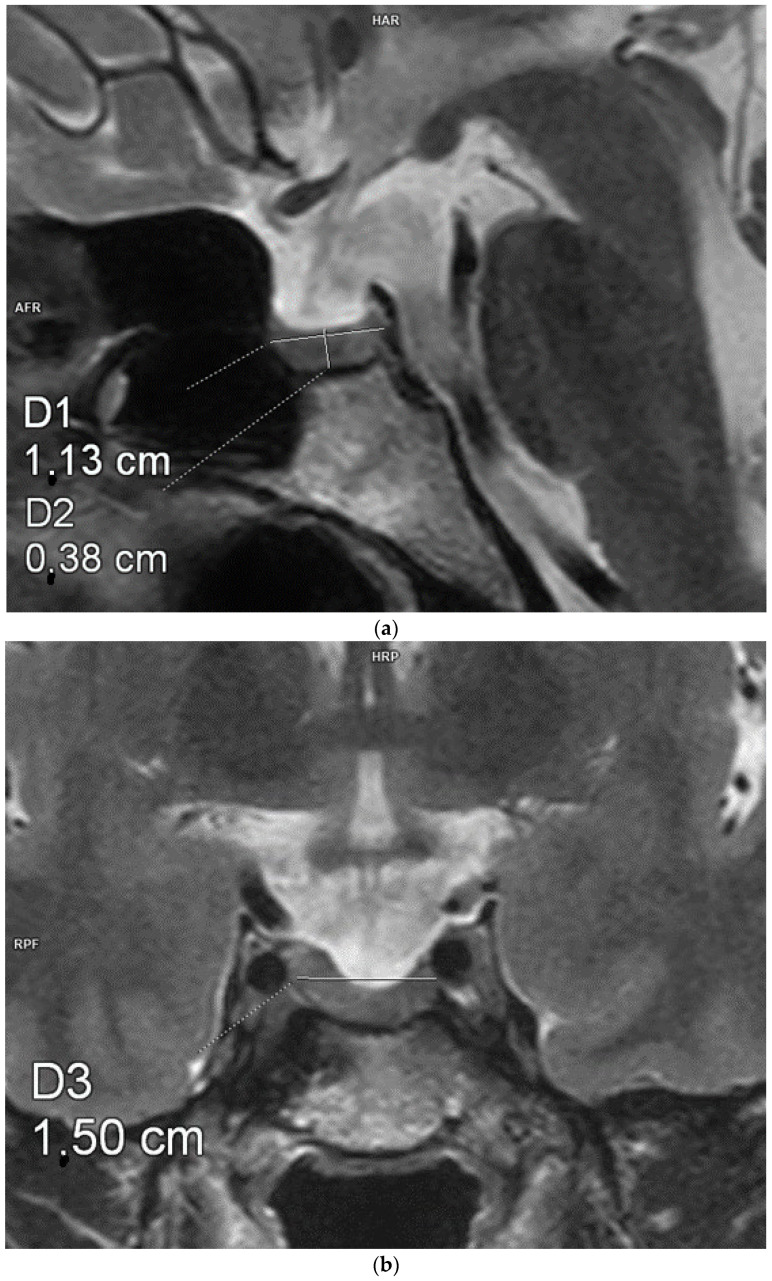
Measurements of the pituitary gland. Magnetic resonance imaging, T2-weighted images in the sagittal (**a**) and coronal (**b**) planes. The craniocaudal, transverse, and anterioposterior dimensions of the pituitary gland are 3.8 mm × 11.3 mm × 15 mm.

Each patient underwent an MRI of the pituitary gland at the Department of Radiological Diagnostics of the National Medical Institute of the Ministry of the Interior and Administration. The examination was performed according to the pituitary protocol, with T1- (+Dixon) and T2-weighted, FLAIR, T2 STIR, and T1-weighted images being recorded after contrast administration using a 3.0 Tesla scanner with a slice thickness of 2 mm, slice gap of 0, field of view 150/150, matrix 240/300, and pixel alimentation of 0.5 × 0.625 mm. The AP, CC, and transverse diameters of the pituitary were measured. The pituitary volume was calculated according to a simplified formula for a rotational ellipsoid (volume = 0.5 × AP diameter × transverse diameter × CC diameter). Figure 1, Figure 2 and Figure 3 show example measurements and calculations of the pituitary volume. The following parameters were also evaluated: pituitary concavity (i.e., its indented appearance) in the coronal and sagittal planes, preserved high signal of the posterior lobe of the pituitary gland in T1-weighted images, anteroposterior or lateral displacement of the pituitary stalk (i.e., infundibular deviation without disruption in its continuity), and suprasellar cistern herniation occupying the upper third or the upper two-thirds of the sella turcica.

Taking into account previous diagnoses, the patients underwent a hormone profile assessment, a fasting blood test at approximately 8:00 a.m. after a night’s sleep, and a morning urine test. In order to assess the function of the anterior pituitary, the following hormone levels were assessed: adrenocorticotropic hormone (ACTH) together with cortisol, growth hormone, insulin-like growth factor 1 (IGF-1), prolactin, thyroid stimulating hormone (TSH) together with free triiodothyronine (fT3) and free thyroxine (fT4), follicle-stimulating hormone (FSH) and luteinizing hormone (LH) together with estradiol or testosterone. An indirect evaluation of posterior pituitary function was performed based on the following parameters: specific gravity of urine, serum and urine osmolality, and serum sodium and potassium levels. Secondary adrenocortical insufficiency was diagnosed based on decreased serum ACTH and cortisol concentrations confirmed with the corticotropin-releasing hormone (CRH) stimulation test. No patient had a history of growth hormone deficiency diagnosed in childhood. Neither the patients’ history nor diagnostic investigations suggested growth hormone deficiency in adulthood. Hyperprolactinemia was diagnosed based on increased prolactin levels measured under standard conditions.

Statistical analysis was conducted with Statistica^®^ software (13.3). Statistical significance was determined with the use of inference tests suitable for the nature and the type of data, i.e., their distribution (verified with the Shapiro–Wilk W test) and variance homogeneity (verified with the Brown–Forsythe’s test). Continuous variables were presented as means ±SD, and the comparisons were made using either the Student’s *t*-test for normally distributed variables with homogeneous variances or the Mann–Whitney U test for dependent variables showing departures from either normal distribution or variance homogeneity. Nominal variables were presented as absolute numbers and percentages and compared using either the chi-square test or the exact Fisher’s test. Spearman’s rank correlation test was used to assess correlations between the pituitary volume and pituitary CC, transverse, and AP diameters. The univariate and multivariate logistic regression model was used to determine the statistical significance of the evaluated variables in predicting hormonal disorders (adrenocortical insufficiency and hyperprolactinemia). Regression results were presented as odds ratio (OR) with a 95% confidence interval (95% CI). In order to minimize the possibility that some outcomes may have been recorded by pure chance due to the low sample sizes of both groups and, additionally, the fact that the diagnosis was incidental, we validated the estimates with the use of resampling techniques. For this purpose, we employed bootstrap-boosted logistic regression analyses (10,000 iterations) using the original sample sizes. *p*-values below 0.05 were considered statistically significant.

The study was approved by the Medical University of Warsaw Bioethical Review Board (no. KB/66/2022) on 26 May 2022. The study was conducted in accordance with the relevant ethical guidelines and regulations. A written informed consent was obtained from each participant.

## 3. Results

No significant differences were observed between the compared groups in terms of variables such as sex, age, body weight, waist circumference, and arterial hypertension. However, patients in Group 2 exhibited a significantly higher mean body mass index (BMI), as shown in Table 1.

Regarding morphological parameters of the pituitary, the compared groups differed in terms of their AP, CC, and transverse diameters, as shown in Table 2. The pituitary glands of patients with an empty sella were characterized by larger AP and transverse diameters and a smaller CC diameter. However, patients with an empty sella ultimately had a significantly smaller pituitary volume compared with that in patients with a partial empty sella. Spearman’s rank correlation test showed a statistically significant positive correlation between pituitary volume and the CC diameter (Spearman R 0.627, *p* = 0.001) as well as the transverse diameter (Spearman R 0.341, *p* = 0.025) and a lack of a significant correlation between pituitary volume and the AP diameter (Spearman R 0.196, *p* = 0.208). Pituitary stalk displacement was more common in patients with an empty sella. Such displacement was both anterior or posterior and lateral; however, only the anterior or posterior component showed statistical significance. Pituitary gland concavity (assessed in the sagittal and coronal planes) and lateral infundibular displacement were more frequently observed in patients with empty sella. However, those differences were not statistically significant.

Hyperprolactinemia was detected in four patients. No significant intergroup differences were observed in terms of hyperprolactinemia. In regard to patients with an empty sella, significantly lower morning ACTH levels were observed, as shown in Table 3. Three patients with empty sella were diagnosed with secondary adrenocortical insufficiency and received hydrocortisone for cortisol replacement therapy. All of these patients had an empty sella on MRI. Additionally, patients with empty sella had a significantly higher BMI than those with partial empty sella syndrome.

**Figure 4:**
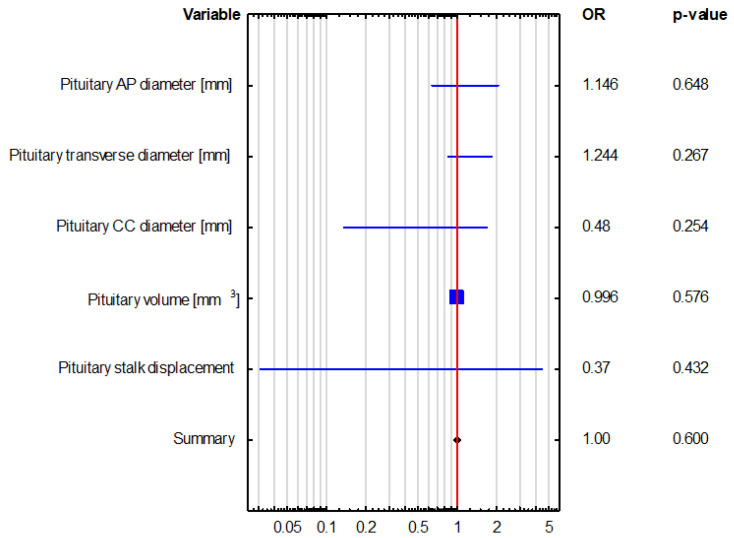
Univariate logistic regression in the assessment of the predictors of adrenocortical insufficiency.

**Figure 5:**
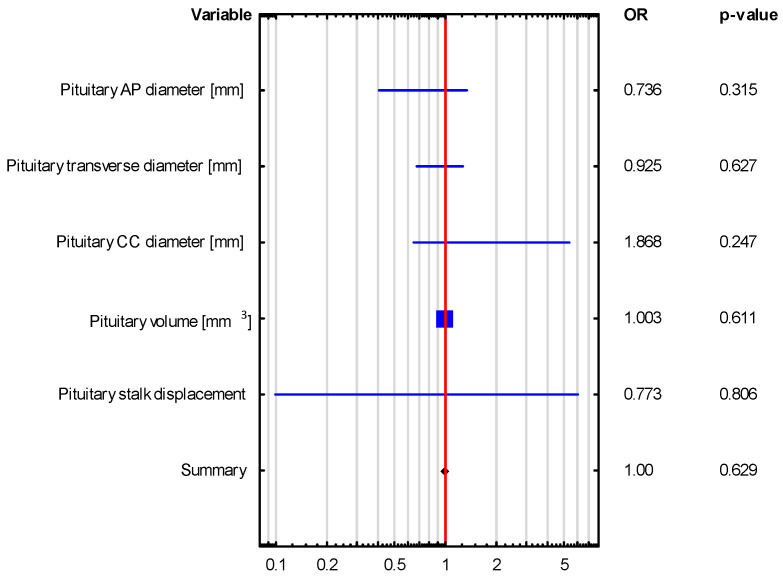
Univariate logistic regression in the assessment of the predictors of hyperprolactinemia.

**Figure 6:**
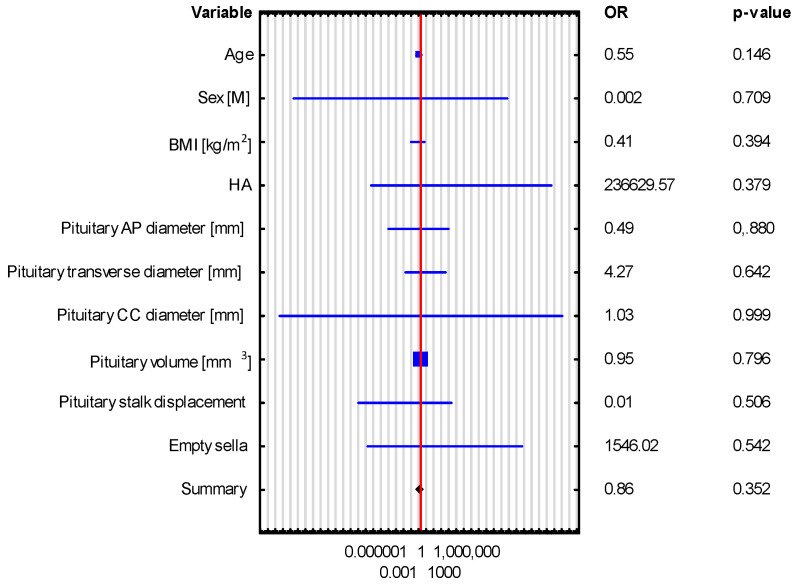
Bootstrap-boosted multivariate logistic regression analysis in the assessment of predictors of adrenocortical insufficiency.

**Figure 7:**
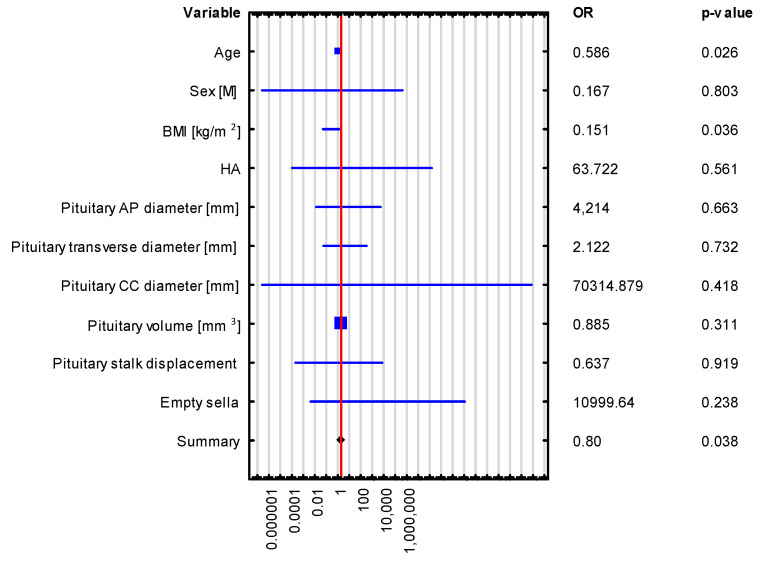
Bootstrap-boosted multivariate logistic regression analysis in the assessment of predictors of hyperprolactinemia.

Univariate logistic regression revealed that pituitary gland dimensions and volume were not significant predictors of hormonal disorders, such as adrenocortical insufficiency and hyperprolactinemia, as shown in Table 4 and Figure 4 and Figure 5. This observation was further confirmed by bootstrap-boosted multivariate logistic regression, adjusted for age, sex, BMI, and hypertension, as shown in Table 5 and Figure 6 and Figure 7. The analysis revealed that age and BMI increase the risk of hyperprolactinemia.

Patients in the compared groups did not significantly differ in terms of estradiol or testosterone levels, even when the sex of the subjects was included in the analysis.

## 4. Discussion

The key radiological features defining partial empty and empty sella syndrome are the pituitary CC diameter and the volume of the subarachnoid space bulging into the sella turcica [1,2,3]. Various definitions of this condition may be found in the literature, which is probably due to the adopted criterion based on the nature and resolution of the diagnostic method. The most common definition of an empty sella—also the one adopted in this paper—is a herniation of the subarachnoid space into the sella turcica occupying at least 50% of its volume and a pituitary height (i.e., its CC diameter) of less than 3 mm; a partial empty sella is defined as a herniation of the subarachnoid space into the sella turcica occupying more than 50% of its volume and a pituitary CC diameter of at least 3 mm [1,2,3,6,7]. In MRI-based research, empty sella was diagnosed when the height of the pituitary was below 3 mm [1,12,14]. In another study based both on MRI and CT scans, empty sella was diagnosed when the height of the pituitary was less than or equal to 2 mm [11]. However, according to the same study, partial empty sella was diagnosed when the height of the pituitary was above 3 mm, without taking into account pituitary heights between 2 and 3 mm [11,12,14]. Other definitions were based on the criterion of the percentage of sellar volume occupied by the bulging suprasellar space, with the most common cut-off point being 50% or 60% [11,13].

In our patients, a significant difference in pituitary volume was observed between the compared groups. However, patients with empty sella ultimately had a smaller pituitary volume compared with patients with partial empty sella. Patients with empty sella exhibited greater AP and transverse diameters and a smaller CC diameter of the pituitary than patients with partial empty sella. We believe that measuring pituitary volume may help in the diagnosis of empty and partial empty sella syndrome. However, it is important to note that pituitary volume shows a positive correlation with pituitary height, which seems to justify the use of the current definition of empty and partial empty sella in everyday clinical practice. Pituitary stalk displacement in the AP plane was observed significantly more frequently in patients with empty sella. Pituitary concavity was also assessed; however, no difference was found between the compared groups in this respect.

Our observations are consistent with reports in the available literature [15,16,17]. An evaluation of the influence of pituitary volume on the development of hormonal disorders in patients with primary empty sella revealed that patients with empty sella had a smaller pituitary volume than either patients with partial empty sella or healthy individuals from the control group (0.23 ± 0.17 vs. 0.35 ± 0.15 vs. 0.54 ± 0.17 cm^3^, *p* = 0.001) [15]. A similar pituitary volume (0.5 ± 0.6 cm^3^) was demonstrated in a study examining postpartum hypopituitarism in patients with empty and partial empty sella [16]. Another study showed empty sella to be more common in elderly patients, who were characterized by a smaller pituitary volume (0.2 ± 0.13 cm^3^) than in young patients (0.4 ± 0.11 cm^3^) [17].

Although secondary adrenocortical insufficiency and lower serum ACTH levels were observed significantly more often in patients with empty sella, logistic regression showed none of the morphological parameters of the pituitary gland (i.e., its volume; AP, CC, or transverse diameters; or stalk displacement) to be a significant predictor of hormonal disorders, such as adrenocortical insufficiency or hyperprolactinemia.

The abovementioned analysis of the impact of pituitary volume on the rates of hormonal disorders in patients with primary empty sella showed that measuring pituitary volume was helpful in the diagnosis of empty and partial empty sella; however, pituitary volume was not correlated with the concentrations of hormones produced by the anterior pituitary [15]. Similarly, a study evaluating the effect of age on pituitary volume revealed no relationship between pituitary volume and the gland’s hormone-secreting function [17]. Notably, certain physiological factors may also affect pituitary volume, but not its secretory function. Studies assessing pituitary volume in patients with no diagnosed pituitary pathologies showed that women aged 50–66 were characterized by a greater volume of the pituitary gland [18,19]. Apart from sex, other factors, such as age and periods of increased hormone production (e.g., puberty and pregnancy), may affect pituitary volume [20,21,22]. A study evaluating radiological prognostic factors for the occurrence of hormonal disorders in patients with empty sella demonstrated that patients with hormonal disorders were characterized by a significantly lower height of the adenohypophysis measured both on the right (1.54 ± 0.84 vs. 1.96 ± 0.83 mm, *p* < 0.05) and left (1.66 ± 0.8 vs. 1.94 ± 0.94 mm, *p* < 0.05) side of the infundibulum than patients without hormonal disorders. Therefore, it was concluded that adenohypophyseal height was a good determiner of hormonal disorders [23]. Some authors also mentioned that pituitary stalk compression and displacement might constitute risk factors for hormonal disorders, especially hyperprolactinemia [9,23,24].

In our study, hyperprolactinemia occurred in four patients (9.3%), with similar rates noted in patients with partial empty and empty sella. Secondary adrenocortical insufficiency was diagnosed in three patients (6.9%) only in the empty sella group. Patients in this group also had significantly lower serum ACTH levels. This result may be partially influenced by the low serum ACTH levels in the three abovementioned patients with clinical adrenal insufficiency and, additionally, seems to be of no clinical importance, as it did not result in lower cortisol levels in patients with empty sella. No other hormonal disorders were observed.

Hyperprolactinemia is the most commonly described hormonal disorder in patients with partial empty and empty sella. Depending on the study, it may affect 10–37.5% of those patients and may be associated with pituitary stalk compression and inhibited dopamine secretion (dopamine levels of 94 ng/mL), and dopamine is a known prolactin secretion inhibitor [9,23,24,25,26,27,28]. The multivariate logistic regression analysis in our study showed BMI and age to be risk factors for hyperprolactinemia in patients with empty and partial empty sella. Overweight and obesity are known risk factors for empty and partial empty sella [3,8,10], and hyperprolactinemia most commonly affects patients aged 20–40 years [28]. Nonetheless, some authors reported no significant differences between patients with obesity (BMI ≥ 30 kg/m^2^) and those without obesity (BMI < 30 kg/m^2^) in terms of prolactin levels (22.21 ± 42.95 vs. 22.22 ± 42.95, *p* = 0.447), pituitary volume (403.2 ± 159.7 vs. 404.2 ± 157.6, *p* = 0.979), or the incidence of empty sella (5.79% vs. 0%, *p* = 0.212); however, reduced pituitary volume was observed in patients with obesity who were unsuccessful in lowering their body weight. These findings encourage further studies in a larger group of patients [29].

Various authors reported that adrenocortical insufficiency might affect up to 37.5% of patients. It was more commonly observed in patients with empty sella than in those with partial empty sella. The pathogenesis of adrenocortical insufficiency may be associated with the location of corticotropic cells in the anterior pituitary. Therefore, they may be more susceptible to compression by the suprasellar cistern [14,26,30]. Patients with empty and partial empty sella may also develop other disorders, such as growth hormone deficiency (8–60%), hypogonadotropic hypogonadism (6–55.9%), secondary hypothyroidism (4.3–54.6%) and diabetes insipidus (9.5%), whereas hypopituitarism was found to be more common in patients with empty sella and, depending on the criterion adopted in the given study, might affect up to 68% of cases [10,30,31,32,33,34].

One limitation of our study is a small sample size; however, the study population was selected via homogenous sampling from a larger population, in which the evaluated condition is uncommon.

In the future, prospective multicenter studies should be carried out in a large group of patients with long follow-up periods.

## 5. Conclusions

The patients from the evaluated study groups differed significantly in terms of pituitary volume. The patients with empty sella (i.e., the CC pituitary diameter of less than 3 mm) exhibited larger transverse and AP pituitary diameters but lower pituitary volume than patients with partial empty sella. We believe that measuring pituitary volume may help in diagnosing empty and partial empty sella syndrome. However, since pituitary volume shows a positive correlation with pituitary height, using the current definition of empty and partial empty sella in everyday clinical practice is justified.

Most of the patients in the examined population with an empty sella or partial empty sella syndrome do not manifest any endocrine pathology. Although hormone disorders were not common in our study population and logistic regression showed empty sella not to be a risk factor for such hormone disorders as adrenocortical insufficiency or hyperprolactinemia, patients with empty sella still exhibited higher rates of secondary adrenocortical insufficiency, which demonstrates that patients with empty and partial empty sella should undergo hormone tests as part of a routine assessment protocol.

This requires more studies in a larger group of patients in order to help develop definitive guidelines.

## Figures and Tables

**Table 1:** Clinical characteristics of patients diagnosed with partial empty sella (Group 1) and with empty sella (Group 2).

VARIABLE	GROUP 1 (*n* = 20)	GROUP 2 (*n* = 23)	*p*-VALUE
Age [years]	54.8 ± 12.4	59.5 ± 16.4	0.271
Sex [M]	6 (30%)	3 (13%)	0.173
Body weight [kg]	76.6 ± 14.55	78.9 ± 13.01	0.588
BMI [kg/m^2^]	25.8 ± 6.5	29.7 ± 4.8	0.039
Waist circumference [cm]	90.7 ± 11.7	94.7 ± 14.1	0.208
Hypertension [*n*, %]	6 (31.6%)	11 (55%)	0.141
Normal thyroid function [*n*, %]	19 (95%)	22 (95.7%)	0.919
Primary hypothyroidism [*n*, %]	4 (20%)	6 (26.1%)	0.522
Hyperprolactinemia [*n*, %]	2 (10%)	2 (8.7%)	0.883
Secondary adrenocortical insufficiency [*n*, %]	0 (0%)	3 (13.04%)	0.046

**Table 2:** Radiologic characteristics of patients diagnosed with partial empty sella (Group 1) and with empty sella (Group 2).

VARIABLE	GROUP 1 (*n* = 20)	GROUP 2 (*n* = 23)	*p*-VALUE
Pituitary AP diameter [mm]	11.4 ± 1.4	13.2 ± 1.9	0.003
Pituitary transverse diameter [mm]	14.8 ± 2.9	17.2 ± 2.9	0.016
Pituitary CC diameter [mm]	3.9 ± 0.62	2.2 ± 0.6	0.001
Pituitary volume [mm^3^]	332.8 ± 107.6	243.5 ± 70.9	0.001
Pituitary concavity	12 (60%)	17 (73.9%)	0.331
Pituitary concavity in the coronal plane	12 (60%)	17 (73.9%)	0.331
Pituitary concavity in the sagittal plane	11 (55%)	17 (73.9%)	0.194
Pituitary stalk displacement	7 (35%)	17 (73.9%)	0.010
Anteroposterior pituitary stalk displacement	7 (35%)	16 (69.6%)	0.023
Lateral pituitary stalk displacement	0 (0%)	3 (13%)	0.094
Upper and middle sella turcica filled with CSF	15 (75%)	21 (91.3%)	0.149

Note. *n* = 20 for group 1 and *n* = 23 for group 2. Abbreviations: AP—anteroposterior, CC—craniocaudal, CSF—cerebrospinal fluid.

**Table 3:** Laboratory test results of patients diagnosed with partial empty sella (Group 1) and with empty sella (Group 2).

VARIABLE	GROUP 1 (*n* = 20)	GROUP 2 (*n* = 23)	*p*-VALUE
Specific gravity of urine [g/mL]	1.01 ± 0.001	1.01 ± 0.01	0.809
Serum sodium [mmol/L]	139.8 ± 3.65	141.3 ± 2.32	0.195
Serum potassium [mmol/L]	4.44 ± 0.38	4.51 ± 0.44	0.538
Serum osmolality [mOsm/kg]	291.3 ± 8.1	289.6 ± 4.9	0.621
Urine osmolality [mOsm/kg]	569.3 ± 215.7	570.4 ± 221.1	0.971
TSH [µIU/mL]	1.83 ± 0.92	2.06 ± 0.96	0.446
fT3 [pg/mL]	3.1 ± 0.4	3.1 ± 0.4	0.609
fT4 [ng/dL]	1.27 ± 0.2	1.23 ± 0.16	0.681
Prolactin [ng/mL]	12.6 ± 10.6	13.9 ± 7.1	0.171
Cortisol at 8 a.m. [µg/dL]	12.9 ± 3.5	14.7 ± 4.5	0.362
ACTH at 8 a.m. [pg/mL]	27.5 ± 13.2	21.8 ± 17.6	0.039
Growth hormone [ng/mL]	2.13 ± 4.1	0.83 ± 1.3	0.461
IGF-1 [ng/mL]	143.6 ± 47.1	152.6 ± 65.5	0.991

TSH—thyroid-stimulating hormone, fT3—free triiodothyronine, fT4—free thyroxine, ACTH—adrenocorticotropic hormone, IGF-1—insulin-like growth factor 1, BMI—body mass index.

**Table 4:** Univariate logistic regression in the assessment of the predictors of adrenocortical insufficiency and hyperprolactinemia.

VARIABLE	Adrenocortical Insufficiency OR (95% CI), *p*-Value	Hyperprolactinemia OR (95% CI), *p*-Value
	The Whole Group *n* = 43	The Whole Group *n* = 43
Pituitary AP diameter [mm]	1.146 (0.639–2.053), *p* = 0.648	0.736 (0.405–1.338), *p* = 0.315
Pituitary transverse diameter [mm]	1.244 (0.846–1.830), *p* = 0.267	0.925 (0.674–1.268), *p* = 0.627
Pituitary CC diameter [mm]	0.48 (0.136–1.694), *p* = 0.254	1.868 (0.649–5.38), *p* = 0.247
Pituitary volume [mm^3^]	0.996 (0.984–1.009), *p* = 0.576	1.003 (0.992–1.013), *p* = 0.611
Pituitary stalk displacement	0.37 (0.031–4.418), *p* = 0.432	0.773 (0.099–6.061), *p* = 0.806

Note. OR, presented as OR (95% CI), was calculated with the aid of univariate logistic regression analysis. Abbreviations: AP—anteroposterior, CC—craniocaudal.

**Table 5:** Bootstrap-boosted multivariate logistic regression analysis in the assessment of the predictors of adrenocortical insufficiency and hyperprolactinemia.

VARIABLE	Adrenocortical Insufficiency OR (95% CI), *p*-Value	Hyperprolactinemia OR (95% CI), *p*-Value
	The Whole Group *n* = 43	The Whole Group *n* = 43
Age	0.552 (0.247–1.231), *p* = 0.146	0.586 (0.365–0.939), *p* = 0.026
Sex [M]	0.002 (4.03245 × 10^−17^–1.39996 × 10^11^), *p* = 0.709	0.167 (1.34 × 10^−7^–208,177.659), *p* = 0.803
BMI [kg/m^2^]	0.411 (0.053–3.179), *p* = 0.394	0.151 (0.025–0.889), *p* = 0.036
HTN	236,629.574 (2.47841 × 10^−7^–2.25925 × 10^17^), *p* = 0.379	63.722 (5.13 × 10^−5^–79,090,557.431), *p* = 0.561
Pituitary volume [mm^3^]	0.954 (0.669–1.360), *p* = 0.796	0.885 (0.699–1.121), *p* = 0.311
Pituitary AP diameter [mm]	0.493 (3.75585 × 10^−5^–6481.229), *p* = 0.884	4.214 (0.006–2734.803), *p* = 0.663
Pituitary transverse diameter [mm]	4.273 (0.009–1957.818), *p* = 0.642	2.122 (0.028–158.944), *p* = 0.732
Pituitary CC diameter [mm]	1.028 (4.06766 × 10^−18^–2.59745 × 10^17^), *p* = 0.999	70,314.879 (1.33 × 10^−7^–3.70876 × 10^16^), *p* = 0.418
Pituitary stalk displacement	0.008 (4.92937 × 10^−9^–12,431.538), *p* = 0.506	0.637 (0.0001–3722.328), *p* = 0.919
Empty sella	1546.021 (8.7857 × 10^−8^–2.72054 × 10^13^), *p* = 0.542	10,999.64 (0.002–56,464,947,364.589), *p* = 0.238

Note: OR, presented as OR (95% CI), calculated with the aid of multivariate logistic regression analysis; crude OR values are optionally adjusted for age, sex, BMI, and hypertension. Abbreviations: AP—anteroposterior, CC—craniocaudal, BMI—body mass index, HTN—hypertension.

## Data Availability

The datasets generated during and/or analyzed during the present study are available from the corresponding author upon reasonable request.

## References

[1] Lundholm M.D., Yogi-Morren D. (2024). A comprehensive review of empty sella and empty sella syndrome. Endocr. Pract..

[2] Prabhat N., Kaur K., Takkar A., Ahuja C., Katoch D., Goyal M., Dutta P., Bhansali A., Lal V. (2024). Pituitary Dysfunction in Idiopathic Intracranial Hypertension: An Analysis of 80 Patients. Can. J. Neurol. Sci..

[3] Rice-Canetto T.E., Carroll P., Reier L., Siddiqi J. (2024). Asymptomatic Empty Sella: A Literature Review and Suggestions for Evaluation in Clinical Practice. Cureus.

[4] Saindane A.M., Lim P.P., Aiken A., Chen Z., Hudgins P.A. (2013). Factors determining the clinical significance of an “empty” sella turcica. Am. J. Roentgenol..

[5] Sage M.R., Blumbergs P.C. (2000). Primary empty sella turcica: A radiological-anatomical correlation. Australas. Radiol..

[6] Carosi G., Brunetti A., Mangone A., Baldelli R., Tresoldi A., Del Sindaco G., Lavezzi E., Sala E., Mungari R., Maria Fatti L.M. (2022). A multicenter cohort study in patients with primary empty sella: Hormonal and neuroradiological features over a long follow-up. Front. Endocrinol..

[7] Sorrentino F.P., Chiloiro S., Giampietro A., Bianchi A., Pontecorvi A., De Marinis L. (2024). Empty sella syndrome: An update. Pituitary.

[8] Chiloiro S., Giampietro A., Bianchi A., Tartaglione T., Capobianco A., Anile C., De Marinis L. (2017). Diagnosis of endocrine disease: Primary empty sella: A comprehensive review. Eur. J. Endocrinol..

[9] Auer M.K., Stieg M.R., Crispin A., Sievers C., Stalla G.K., Kopczak A. (2018). Primary Empty Sella Syndrome and the Prevalence of Hormonal Dysregulation. Dtsch. Arztebl. Int..

[10] Guitelman M., Garcia Basavilbaso N., Vitale M., Chervin A., Katz D., Miragaya K., Herrera J., Cornalo D., Servidio M., Boero L. (2013). Primary empty sella (PES): A review of 175 cases. Pituitary.

[11] Zuhur S.S., Kuzu I., Ozturk F.Y., Uysal E., Altuntas Y. (2014). Anterior pituitary hormone deficiency in subjects with total and partial primary empty sella: Do all cases need endocrinological evaluation?. Turk. Neurosurg..

[12] Lupi I., Manetti L., Raffaelli V., Grasso L., Sardella C., Cosottini M., Iannelli A., Gasperi M., Bogazzi F., Caturegli P. (2011). Pituitary autoimmunity is associated with hypopituitarism in patients with primary empty sella. J. Endocrinol. Investig..

[13] Cannavò S., Curtò L., Venturino M., Squadrito S., Almoto B., Narbone M.C., Rao R., Trimarchi F. (2002). Abnormalities of hypothalamic-pituitary-thyroid axis in patients with primary empty sella. J. Endocrinol. Investig..

[14] Colao A., Cotta O.R., Ferone D., Torre M.L., Ferraù F., Di Somma C., Boschetti M., Teti C., Maria CSavanelli Alibrandi A., Trimarchi F. (2013). Role of pituitary dysfunction on cardiovascular risk in primary empty sella patients. Clin. Endocrinol..

[15] Akkus G., Sözütok S., Odabaş F., Onan B., Evran M., Karagun B., Sert M., Tetiker T. (2021). Pituitary Volume in Patients with Primary Empty Sella and Clinical Relevance to Pituitary Hormone Secretion: A Retrospective Single Center Study. Curr. Med. Imaging.

[16] Fleckman A.M., Schubart U.K., Danziger A., Fleischer N. (1983). Empty sella of normal size in Sheehan’s syndrome. Am. J. Med..

[17] Terano T., Seya A., Tamura Y., Yoshida S., Hirayama T. (1996). Characteristics of the pituitary gland in elderly subjects from magnetic resonance images: Relationship to pituitary hormone secretion. Clin. Endocrinol..

[18] Grams A.E., Gempt J., Stahl A., Förschler A. (2010). Female pituitary size in relations to age and hormonal factors. Neuroendocrinology.

[19] Berntsen E.M., Haukedal M.D., Häberg A.K. (2021). Normative data for pituitary size and volume in the general populatin between 50 and 66 years. Pituitary.

[20] Atmaca M., Baykara S., Mermi O., Yildirim H., Akaslan U. (2016). Pituitary volumes are changed in patients with conversion disorder. Brain Imaging Behav..

[21] Pepe J.S., Brouwer R.M., van Leeuwen M. (2010). HPG—axis hormones during puberty: A study on the association with hypothalamic and pituitary volumes. Psychoneuroendocrinology.

[22] Simmons J.G., Whittle S.L., Patton G.C. (2014). Study protocol: Imaging brain development in the Childhood to adolescence Transition study (iCATS). BMC Pediatr..

[23] Atci I.B., Yilmaz H., Karagoz Y., Kocak A. (2018). Prognosis of Hormonal Deficits in Empty Sella Syndrome Using Neuroimaging. Asian J. Neurosurg..

[24] Ekhzaimy A.A., Mujammami M., Tharkar S., Alansary M.A., Al Otaibi D. (2020). Clinical presentation, evaluation and case management of primary empty sella syndrome: A retrospective analysis of 10-year single-center patient data. BMC Endocr. Disord..

[25] De Marinis L., Bonadonna S., Bianchi A., Maira G., Giustina A. (2005). Primary empty sella. J. Clin. Endocrinol. Metab..

[26] Del Monte P., Foppiani L., Cafferata C., Marugo A., Bernasconi D. (2006). Primary “empty sella” in adults: Endocrine findings. Endocr. J..

[27] Leca B.M., Mytilinaiou M., Tsoli M., Epure A., Aylwin S.J.B., Kaltsas G., Randeva H.S., Dimitriadis G.K. (2021). Identification of an optimal prolactin threshold to determine prolactinma size using receiver perating characteristic analysis. Sci. Rep..

[28] Alyami N., Alhenaki G., Al Atwah S., Alhenaki N., Smaisem F., Alotaibi A., Abu Risheh j Smaisem M., Alhenaki A., Alanazi S., Alshammeri M. (2025). Correlation between MRI findings of pituitary gland and prolactin level among hyperprolactinemia adult female Saudi patients in rural areas: A retrospective multicentric study. Medicine.

[29] Puliani G., Sbardella E., Cozzolino A., Sada V., Tozzi R., Andreoli Ch Fiorelli M., Di Basi C., Corallino D., Bala A., Paganini A.M. (2023). Pituitary T1 signal intensity at magnetic resonance imaging is reduced in patients with obesity: Results from the CHIASM study. Int. J. Obes..

[30] Chiloiro S., Giampietro A., Bianchi A., De Marinis L. (2021). Empty sella syndrome: Multiple endocrine disorders. Handb. Clin. Neurol..

[31] Gasperi M., Aimaretti G., Cecconi E., Colao A., Di Somma C., Cannavò S., Baffoni C., Cosottini M., Curtò L., Trimarchi F. (2002). Impairment of GH secretion in adults with primary empty sella. J. Endocrinol. Investig..

[32] Foresti M., Guidali A., Susanna P. (1991). Primary empty sella. Incidence in 500 asymptomatic subjects examined with magnetic resonance. Radiol. Med..

[33] Ran Ch Peng G., Shen R., Liao Q., Wang Q., Zhou L., Zheng H., Long M. (2024). Efficacy of GnRH Pulses in Hypogonadism secondary to Primary Empty Sella: Case report. Reprod. Sci..

[34] Belay K.E., Jemal R.H., Kebede A.H., Tulu M.G., Belay A.E., Haile A.M., Demisse S.A. (2024). Arginine vasopressin deficiency (central diabetes insipidus) with partial empty sella: A case report. BMC Endocr. Disord..

